# Preferences for Long-Acting and Alternative Modalities for PrEP among Military Men Who Have Sex with Men: Segmentation Results of an Adaptive Choice-Based Conjoint Analysis Study

**DOI:** 10.1007/s11524-022-00615-9

**Published:** 2022-03-22

**Authors:** Jose I. Gutierrez, David Vlahov, Alex Dubov, Frederick L. Altice

**Affiliations:** 1 Yale School of Nursing, 400 West Campus Drive, Orange, CT 06477 USA; 2 National Clinician Scholars Program, University of California, San Francisco, 3333 California St., San Francisco, CA 94118 USA; 3 Department of Epidemiology-Microbial Diseases, Yale School of Public Health, 60 College St, New Haven, CT 06510 USA; 4 Loma Linda University, Griggs Hall 11065, Loma Linda, CA 92350 USA; 5 Department of Medicine, Section of Infectious Diseases, Yale School of Medicine, 333 Cedar Street, New Haven, CT 06510 USA

**Keywords:** Conjoint analysis, Pre-exposure prophylaxis, PrEP, LAI-PrEP, Patient Preferences, Decision science, HIV, Military health, Infectious disease

## Abstract

The use of pre-exposure prophylaxis (PrEP) for HIV prevention within the U.S. military is low. Implementing preference-based alternative modalities of PrEP delivery, however, can be an innovative strategy to address the specific barriers to PrEP uptake among military MSM. We sought to identify population-based, segment-specific preferences for longer-acting and alternative PrEP delivery modalities to guide patient-centered strategies to optimize uptake within military-serving healthcare systems. HIV-negative military men who have sex with men (MSM) completed an anonymous, adaptive choice-based conjoint (ACBC) analysis survey consisting of five key attributes of interest (dosing method, provider type, visit location, lab work evaluation location, and dispensing venue). Relative importance and part-worth utility scores were generated using Hierarchical Bayes (HB) estimation, and cluster ensemble analysis grouped participants into “phenotype” segments by preference similarity. The randomized first-choice model was then used to examine changes in program interest rates among segments through market simulation. The 429 participants were segmented into five preference groups. The dosing method attribute was found to be the most important to nearly all segments. Simulations revealed that PrEP program interest among two segments with low interest levels increased when smartphone, civilian-based, and long-acting injectable PrEP options were involved. Findings also suggested a need for clinics to be responsive and sensitive to sexual practices, risk perception, and functional PrEP knowledge. Responsiveness to segment-specific preferences in the design of military PrEP programs and acting on the importance of clinical relationships within the context of PrEP engagement within a military setting may contribute to increasing PrEP uptake.

## Introduction

Within the U.S. military, there are approximately 350 new HIV infections diagnosed each year; disproportionally affecting those who are younger, Black, and men who have sex with men (MSM) [1–5]. The use of pre-exposure prophylaxis (PrEP) effectively prevents HIV infection [6–9], and PrEP-related services and medications are currently covered by the Department of Defense’s TRICARE medical insurance program [10]. Yet a military pharmacy analysis reveals that only 2000 of the estimated 12,000 PrEP-eligible individuals in the military have accessed PrEP, with access primarily based on the member’s geographic location relative to a military PrEP-prescribing facility [2]. Military MSM have experienced distinct barriers to PrEP delivery and HIV preventative services when serving within the military healthcare system, such as restricted healthcare access [11], heightened stigma [11, 12], and disclosure discomfort [13]. This low uptake of PrEP, however, cannot be attributed to a lack of interest, as the broader literature has demonstrated high levels of interest and acceptability to a variety of PrEP delivery solutions [14–17]. Therefore, innovative strategies that can guide the rapid scale-up of PrEP services that are tailored to address the unique needs and challenges of military MSM are needed within the U.S. military healthcare system. It has been shown that health services that match individual treatment preferences positively affect treatment outcomes and uptake [18–20]. Thus, our objective was to identify the most preferred attributes of alternative PrEP delivery modalities that are most likely to influence uptake among this population.

Stated preference methods, such as conjoint analysis, are market research strategies particularly suited to quantify consumer preference data of new market products and programs, including for PrEP [17, 21–27]. Originating from mathematical psychology, the foundational theory of conjoint analysis is that consumers view products and interventions as a composition of various attributes and will place a certain amount of value (part-worth utility score) on each of the attributes. These values can then be entered into market simulation models to predict how consumers might respond to *any* potential combination of attribute levels [17, 21, 23–27].

While examining utility scores can provide many insights on population-level preferences, large full-sample data can sometimes mask subtle or hidden preference relationships between smaller subgroups of interest—i.e., segments [28]. Therefore, segmenting respondents into homogenous clusters based on similar preferences can reveal nuanced preference data for alternative PrEP delivery models that differ from standard, currently available PrEP programs [29–31]. While PrEP is most commonly available as a daily tablet regimen [6–9], its efficacy can be reduced by pill fatigue, disclosure discomfort, and non-adherence [32, 33]. Thus, alternative modalities of PrEP have been explored to address the barriers that affect the uptake and adherence to a daily tablet regimen. Studies examining tablet regimens on an intermittent, “on-demand” dosing schedule have demonstrated high efficacy among men who have sex with men [34, 35]. Additionally, new Phase III trials of long-acting, injectable formulations of PrEP (HPTN 083/084) found them to have a superior protective effect against HIV acquisition compared to daily oral PrEP among cisgender MSM and transgender women [36], as well as cisgender women [37]. Furthermore, the feasibility and acceptability of conceptual PrEP modalities through implants and rectal douches have also been explored [38–41]. As long-acting injectable (LAI-PrEP) and alternative modalities of PrEP delivery transition towards real-world implementation, it will be crucial to characterize how U.S. military MSM cluster by preferences for various PrEP delivery programs.

## Methods

Between March and April 2020, we partnered with a non-profit organization that supports the needs of LGBTQ military and veteran members to recruit U.S. military MSM and trans-individuals through a closed online social media group comprised of over 7000 self-reported LGBTQ U.S. military members [42]. Organization administrators disseminated weekly advertisements for the study within the group, and interested participants could access the anonymous survey with a “click to consent” procedure if eligible. Participants were provided an option to receive $5 compensation for questionnaire completion, and the study was approved by the Yale University Institutional Review Board.

Clustering participants by preferences first begins with identifying individual-level part-worth utilities from a conjoint experiment [29–31]. With a focus on modifiable PrEP program attributes and levels, an adaptive choice-based conjoint (ACBC) analysis survey was developed based on a review of the literature and in-depth, qualitative interviews from PrEP experts and active-duty military MSM [2–5, 11–13, 34, 39, 40, 43–53]. The final survey design was composed of five different PrEP program delivery attributes (and associated levels) that included **dosing method** (*daily tablet, on-demand [before sex] tablet regimen, rectal douche [before sex], injection [every 2 months], implant [once a year]*), **provider type** (*military, civilian*), **visit location** (*on-base, off-base, smartphone app*), **dispensing venue** (*on-base, off-base, mail delivery*), and **lab evaluation** (*on-base, off-base, home-based mail-in kit*). Before implementation, the survey was piloted and revised with 11 military MSM to ensure that attributes and associated levels were understood, logical, and relevant to a military PrEP program. Additional demographical data was also collected to include age, race, ethnicity, rank type, military branch, geographic region, PrEP experience (“Have you ever used PrEP [Pre-Exposure Prophylaxis]?”), HIV protection satisfaction level, disclosure discomfort, and the HIV Incidence Risk Index for MSM (HIRI-MSM) score [54].

## Analysis

The final survey instrument was loaded into Lighthouse Studio 9 and pre-tested for choice task configuration. To achieve a high degree of individual-level precision, the final survey design displayed each attribute level to respondents at least 3 times, had a standard of error of < 0.03, and reported efficiencies were all 1.000 [55].

Multiple quality control strategies were employed to protect data integrity. First, security features within the Sawtooth software prevent repeat survey submissions through internet browser cookies and IP addresses [56]. Next, extensive pilot testing revealed that the survey could not be taken in less than 10 to 15 min, thus omitting responses completed in less than 10 min. Additionally, the root likelihood (RLH) fit statistic for each respondent was analyzed to evaluate whether respondents that answered choice questions consistently or randomly at the 95% percentile (0.5178 RLH) [57]. Thus, survey respondents with an RLH below this score were omitted to ensure a 95% confidence level that random responders will fall below this cut-off level [57].

Data analysis began with generating descriptive statistics of the aggregate sample, to include frequencies and measures of central tendency. For conjoint data, part-worth utility scores of all 16 attribute levels were generated using Hierarchical Bayes (HB) estimation [58, 59]. The resulting part-worth utility scores are zero-centered, meaning that scores that are further away from zero indicate a stronger positive or negative preference for the level choice in relation to the other level choices under the same attribute [53, 55, 59]. The attribute relative importance scores, which reflect the amount of influence each attribute has on the respondent’s decision-making, were also calculated by dividing the range of part-worth utility scores for each attribute by the sum of the ranges and multiplying by 100 [60, 61].

Next, cluster ensemble analysis was used to group respondents by individual preferences. For this study, we utilized the “K-Means” method and performed 30 replications with mixed starting points [29]. A five-group cluster solution was achieved with a reproducibility of 82.4% within all clustering algorithms. Reproducibility standard norm within ensemble analysis for a five-segment cluster solution with 10 to 20 basis variables is 76% [30], indicating that this final five-group cluster solution of 82.4% reproducibility was robust and of high validity [29]. Non-parametric tests were then applied to identify significant variance between segments.

Finally, each group’s part-worth utility scores were used to predict the share of preference (program interest rates) to several combinations of PrEP program scenarios through market simulations. Program interest rates for these PrEP scenarios were generated utilizing the randomized first-choice model [60, 61]. This approach assumes that respondents or consumers will prefer a product with the highest composite part-worth utility score (or value), adjusting for both attribute and program variability [60]. All data analyses were performed using XLSTAT and Sawtooth Lighthouse Studio 9.0.

## Results

### Participants

Tables 1 and 2 in the Appendix display the descriptive statistics of the 429 respondents by preference group. The mean age was 30 years old and 96.7% identified as cis-gendered male. Overall, participants were mostly white (72%), non-Hispanic (72.5%), of officer rank (46.4%), had at least a bachelor’s degree or above (54.1%), and were within the U.S. Army branch (48.7%). Among the aggregate sample, 89.3% received HIRI-MSM scores that defined them as having a high objective risk for acquiring HIV [54]. Cluster ensemble analysis generated five phenotype groups that clustered by preferences and revealed a variety of statistically significant differences. Each phenotype was then labeled with a descriptor based on a defining value.

Group 1 members (*N* = 156, 36.3%), the largest group, tend to have a higher frequency of condomless, receptive anal sex (CRAS) than their peers, with 88.4% of members reporting an episode of CRAS within the past 6 months, and 33.3% reporting CRAS at a frequency of once a week or more. They are also less likely to report consistent condom use (“every time” or “often”) with regular or casual partners within the last 6 months and are the least likely to have had previous use or experience with PrEP. Additionally, they are more likely to feel uncomfortable discussing their sex life with their primary care provider (PCP) (“extremely” or “somewhat” uncomfortable, 39.1%), and are more likely to have a high degree of anticipated stigma from their PCP (“very” or “somewhat” fearful of judgement from their PCP, 48.7%). This group can be labeled [Least PrEP Experience].

Group 2 members (*N* = 42, 9.8%) are more racially and ethnically diverse with a membership that is 38.1% non-white race and 45.2% Hispanic ethnicity, and are most likely to report higher levels of education (“bachelors” or “graduate/professional” degree, 69%). They have more members who report being “tops” (anal insertive position preference, 38.1%), are least likely to have engaged in CRAS within the past 6 months (“none,” 35.7%), and are more likely than their peers to report consistent condom use (“every time” or “often”) with regular and casual partners within the last 6 months. While they are more likely to be satisfied with their current level of HIV protection (97.6%), they are also more likely to feel uncomfortable discussing their sex life with their PCP (“extremely” or “somewhat” uncomfortable, 57.1%). This group can be labeled [Least Comfortable Discussing Sex].

Group 3 members (*N* = 106, 24.7%) tend to be white (82.1%), are more likely to report an education level below a bachelor’s degree (“high school” or “associate degree or some college,” 51.9%), and are more likely to be stationed in the Southern region of the U.S.A (50%). They also tend to prefer anal receptive positions (“versatile” or “exclusively/more bottom,” 84.9%) and are more likely to have had at least one episode of CRAS within the last 6 months (92.5%). Compared to their peers, they are less likely to report consistent condom use with their regular and casual partners within the last 6 months. These members tend to report higher levels of satisfaction with their current level of HIV protection yet are also less likely to have previous use or experience with PrEP. While these members tend to be more comfortable in discussing their sex life with their PCP (“mostly” or “extremely” comfortable, 66.1%), they are also more likely to fear being judged by their PCP for being MSM (“very” or “somewhat” fearful, 60.4%). This group can be labeled [Most Condomless Sex].

Group 4 members (*N* = 91, 21.2%) tend to report an education level below a bachelor’s degree (“high school” or “associate degree or some college,” 53.9%). While these members report higher levels of previous PrEP experience and are less fearful of judgement from their PCP, they are also less likely to be satisfied with their current level of HIV protection. This group can be labeled [Less HIV Protection Satisfaction].

Group 5 members (*N* = 34, 7.9%) are more likely to be stationed in the Northeast region of the U.S.A (38.2%), have the highest membership of individuals who identify as Black (26.5%), and are more likely to report an education level of at least a bachelor’s degree (61.8%). They are also more likely to prefer anal receptive positions (“versatile” or “exclusively/more bottom,” 76.5%), are less likely to have engaged in CRAS within the past 6 months (“none,” 26.5%), and are more likely to report consistent condom use (“every time” or “often”) with regular and casual partners within the last 6 months. Out of all groups, these members report the most experience with PrEP (97.1%) yet are also least likely to be satisfied with their current level or HIV protection (73.5%). Compared to their peers, Group 5 members tend to be more comfortable in discussing their sex life with their PCP and are less fearful of being judged by their PCP for being MSM. This group can be labeled [Most PrEP Experience].

### Relative Importance Scores and Part-Worth Utilities

Tables 3 and 4 in the Appendix show the relative importance and part-worth utility scores of the five attributes stratified by group. Within this population, *dosing method* was the most important attribute for Groups 1, 3, 4, and 5, and the *provider type* was the most important attribute for Group 2.

Group 1 [Least PrEP Experience] had a near equal preference for the PrEP implant and LAI-PrEP and had the strongest preference to see a military provider through a smartphone app for their visits. This large group represents military MSM who want the least demands on their time in terms of taking medications or seeing a provider in person. Group 2 [Least Comfortable Discussing Sex] also strongly preferred the PrEP implant option but were even more influenced by the *provider type* attribute, with a strong preference to see a civilian healthcare provider in an off-base location for their PrEP visits instead. Group 3 [Most Condomless Sex] had the strongest preference for the daily tablet option and also preferred on-base locations for the other aspects of PrEP services. Group 4 [Less HIV Protection Satisfaction] also preferred on-base locations for PrEP services, but instead preferred the on-demand tablet regimen within the *dosing method* attribute. Finally, Group 5 [Most PrEP Experience] most strongly preferred the rectal PrEP douche option with a civilian provider preference in an off-base location, yet also preferred to provide labs and receive PrEP medication in a location on-base.

A separate “none” parameter score was also calculated for each of the five-segment groups through respondent selections within the screening task portion of the ACBC survey. The “none” score represents how likely an individual will select “none” or no PrEP program option versus the hypothetical PrEP program scenarios offered within the exercise. Thus, members who possess a higher or positive “none” score will require a PrEP program to possess attributes that are more closely aligned with their preferences before indicating program interest when compared to individuals with a lower or negative “none” score [55]. Within this study, members within Group 2 [Least Comfortable Discussing Sex] had the highest “none” score, and Group 3 respondents [Most Condomless Sex] had the lowest “none” score.

### Preferences for PrEP Programs

Tables 5 and 6 in the Appendix present the descriptions and share of preference (program interest) rates of the eight PrEP program scenarios that were constructed to examine program interest among the five groups. Table 7 in the Appendix displays the cross-elasticity effects in program interest rates when offering multiple PrEP program options at once within market simulations.

Differences in program interest rates are observed when multiple program scenarios are offered together as available options, as opposed to being offered as a single choice. Scenario 1 (On-Base Military Daily Tablet) best represents the current policy and structure of a standard, daily tablet PrEP program within the military healthcare system [2, 62]. When this PrEP scenario is offered alone, it has an overall total sample program interest rate of 66.4%, with higher program interest rates among Groups 1, 3, and 4 (71.1%, 92.2%, and 69.1%, respectively) and substantially lower program interest rates among Groups 2 and 5 (2.9% and 35.4%, respectively). In a hypothetical market simulation where Scenario 1 (On-Base Military Daily Tablet) was offered in addition to Scenario 2 (Smartphone Military Daily Tablet) and Scenario 6 (Civilian Daily Tablet), then the overall program interest rate increases to 81.1% among the aggregate sample [PrEP Program Grouping #1, Table 7 in the Appendix], with significant gains in program interest rates among Groups 2 and 5 (64.9% and 52.9%, respectively). Overall program interest rates are further increased to 90.6% among the aggregate sample [PrEP Program Grouping #2, Table 7 in the Appendix] when LAI-PrEP (Scenario 4, Smartphone Military Injection) and implants (Scenario 5, Smartphone Military Implant and Scenario 7, Off-Base Civilian Implants) are also accessible in addition to military- and civilian-based daily tablet PrEP programs. When offering the daily tablet and LAI-PrEP programs together, overall program interest is increased from substantial new member gains occurring within Group 1 [Least PrEP Experience], Group 2 [Least Comfortable Discussing Sex], and Group 5 [Most PrEP Experience]. Most program trade-off within these three groups occurred from members shifting away from daily tablet PrEP programs to the newly introduced scenarios featuring LAI-PrEP and implants.

## Discussion

This study’s major findings reveal five unique segments, or phenotypes, within U.S. military MSM with distinct differences among key preference attributes, demographics, sexual practices, PrEP experience, protection satisfaction, provider-related fear, and disclosure discomfort that can significantly influence interest with a PrEP program. Results from the cluster analysis and market simulation demonstrates that maximizing PrEP engagement among segments with lower PrEP program interest must move beyond a “one-size-fits-all” approach and prioritize the availability of additional LAI-PrEP and implant delivery programs. Additionally, segment-specific discomfort and provider-related fear underlines the importance of clinical relationships within a PrEP delivery program. The demonstrated preference for PrEP engagement through different, non-military settings (i.e., civilian provider, off-base setting) within two segments (Groups 2 and 4) compels us to speculate whether the preference to access PrEP through alternative means is a marker of irretrievable distrust of the clinical environment. Either way, the data suggest that the importance of clinical relationships, the design of programs that are responsive to group-specific preferences, and the widest flexibility of options should command more attention.

Overall, the data suggest that the most important factors to consider when designing a PrEP delivery program for military MSM are the *dosing method* for Groups 1, 3, 4, 5 and *provider type* for Group 2. While individuals varied in preferences within other attributes, the highest increase in segment-specific PrEP program interest rates occurred when scenarios featured the PrEP implant for Group 1 [Least PrEP Experience], the civilian healthcare provider for Group 2 [Least Comfortable Discussing Sex], the daily tablet for Group 3 [Most Condomless Sex], the on-demand regimen for Group 4 [Less HIV Protection Satisfaction], and the rectal PrEP douche for Group 5 [Most PrEP Experience]. Out of all the groups, members in Group 2 possess the highest “none” utility scores, which means that these individuals are less likely to express an interest in a PrEP program scenario. This is relevant as the preferred PrEP delivery characteristics for this group—a civilian provider and the PrEP implant—are not likely to be widely available for all military members in the near future. These preferences may be explained by higher levels of discomfort when discussing sex among Group 2 members, but ultimately these preferences result in lower program interest rates within scenarios that tend to do best within the aggregate population (e.g., Scenario 2, Smartphone Military Daily Tablet). In contrast, members of Groups 1 and 3 are less likely to be sensitive to incongruence between PrEP program attributes and their preferences, with most of these individuals reporting higher program interest rates to a wider variety of program configurations.

The data also show that augmenting standard military daily tablet PrEP programs with additional PrEP program options that use a smartphone app and civilian providers will reduce clinical demands on patients and increase the overall program interest rate by over 11%. Total program interest rates can be increased even further by offering members LAI-PrEP and implant options through military and civilian platforms (Table 7 in the Appendix). Utilizing smartphone and telehealth strategies is a particularly salient strategy given the limited access to PrEP that service members face today. Currently, PrEP access within the military healthcare system is uneven and appears mostly dependent on geographic proximity to a major military medical center [63]. Accessing PrEP through smartphones or telehealth to more remote areas could markedly increase access. Previous research, however, has shown that telehealth strategies have successfully circumvented geographic challenges to expand access to expert consultation [64], and that distance-based and home, self-testing options are highly accepted and in-demand for future use [63]. Additionally, the use of LAI-PrEP and implants may be a suitable answer for military members with an unpredictable work or deployment schedule that can inhibit adherence to the medication and follow-up requirements of a standard daily tablet PrEP regimen. As LAI-PrEP and implants move from clinical phase trials to routine practice [41, 65–67], the high level of interest for these LAI-PrEP agents suggests a substantial potential market for improved, future PrEP engagement among military MSM. Therefore, key stakeholders and policymakers within the Department of Defense should make a concerted effort to expand access to PrEP-prescribing medical facilities through the use of smartphone and telehealth visits, and to incorporate the use of LAI-PrEP and implants as they become available.

Descriptive statistics highlight several significant differences between groups that warrant exclusive assessment. Group 1 [Least PrEP Experience] and Group 3 [Most Condomless Sex] are less likely to have previous use or experience with PrEP, less likely to consistently use condoms with regular and casual partners, and report the highest amounts of condomless receptive anal sex out of all groups. Groups 1 and 3 members are also more likely to fear being judged for their gay/MSM identity by their PCP, which is not an unfounded finding given the history of *Don’t Ask, Don’t Tell* (DADT), a since-repealed policy that previously discharged military members for disclosing a same-sex orientation [68]. Despite this heightened fear of anticipated stigma, members in both Groups 1 and 3 also have strong preferences for a PrEP program that includes a visit with a military provider. This finding suggests an opportunity for further research to investigate if the fear of anticipated stigma from a PCP is related to military providers on-base or to civilian providers off-base as Group 3 members are more likely to be stationed in the South; a region historically known for lower PrEP uptake and higher HIV-related stigma [69, 70]. Regardless, the combination of higher frequency of CRAS, less consistent use of condoms, and lower previous experience with PrEP can place these members among those with the highest risk for acquiring HIV [54]. Thus, a priority should be made to ensure that these higher risk members have seamless access to PrEP services within the military healthcare system that are responsive to their preferences, as well as healthcare visits that are respectful and non-judgmental to sexual practices and behaviors to further enhance the interaction experience. While members in Group 2 [Least Comfortable Discussing Sex] and Group 5 [Most PrEP Experience] report higher levels of education, are less likely to engage in CRAS, and are more likely to report consistent condom use with regular and casual partners, they both report HIRI-MSM scores that define the majority of their members as having high objective risk for acquiring HIV [54]. Literature on self-perceived risk and PrEP uptake among at-risk individuals continues to expand, with uptake impacted by factors that include beliefs on medication side-effects [71], low perceived personal risk [72], and low PrEP functional knowledge [73]. Therefore, strategies to increase PrEP uptake should include assessing members to determine if barriers or resistance to uptake are rooted in modifiable knowledge and beliefs. With respect to PrEP experience and HIV satisfaction levels, members in Group 4 [Less HIV Protection Satisfaction] and Group 5 both report the highest proportion of previous PrEP use, yet also the lowest amount of satisfaction with their current level of HIV protection. As members within Groups 4 and 5 have dominating preferences for alternative PrEP dosing methods, such as on-demand regimens, PrEP implants, and rectal douches, further research should explore if a member’s satisfaction with their current level of HIV protection is associated with a preferred PrEP delivery model that may not yet be currently available.

Despite the many new and important findings, this study is not without  limitations. The anonymous survey relied on self-reported measures for participation eligibility, and thus we must consider the potential for respondent bias as actual military status of the participants cannot be confirmed. As this study identifies and reports on preference data only, future studies will need to examine how preference data translates into actual behavior once implemented into practice. As there is no real “lived” experience with some PrEP delivery options (e.g., implants, rectal douche), interest in these attributes may change as more individuals report on their satisfaction and safety. Additionally, the survey was drawn from a convenience sample recruited from an online social media group, which raises concerns about generalizability and responder bias. Military members who participate in this online LGBT social media group may be more forthcoming with their sexual identity and preferences. Hence, these findings may not be generalizable to at-risk military members who do not identify as being MSM or LGBT.

## Conclusion

Preferences and interest for PrEP by at-risk U.S. military service members differ by subgroups, all of which could benefit from PrEP if its delivery were aligned with patient preferences. Five preference groups emerged and suggested that PrEP uptake can be optimized when PrEP delivery programs are designed around the diverse preferences among this population. Models predicted the extent to which the addition of distinct options could translate to a higher level of interest for PrEP engagement. These data can be informative in tailoring the design of PrEP programs to lower participating groups, ultimately improving the overall uptake rate of PrEP within the U.S. military population.

## Data Availability

The datasets used and/or analyzed during the current study are available from the corresponding author on reasonable request.

## Appendix

Tables 1, 2, 3, 4, 5. 6, and 7.

**Table 1:** Characteristics of the participant demographics, segmented by group

Variable	Total (*n* = 429)	Least PrEP Experience [Group 1] (*n* = 156)	Least Comfortable Discussing Sex [Group 2] (*n* = 42)	Most Condomless Sex [Group 3] (*n* = 106)	Less HIV Protection Satisfaction [Group 4] (*n* = 91)	Most PrEP Experience [Group 5] (*n* = 34)	*F*/*χ*^2^ (*p*-value)
*Age:* mean (± SD)	29.9 (± 4.7)	29.9 (± 4.9)	31.3 (± 5.1)	29.8 (± 4.7)	29.5 (± 4.4)	30.0 (± 3.2)	0.33
*Gender*							0.12
Male	415 (96.7%)	153 (98.1%)	42 (100%)	98 (92.5%)	89 (97.8%)	33 (97.1%)	
Trans female	11 (2.6%)	3 (1.9%)	0 (0%)	5 (4.7%)	2 (2.2%)	1 (2.9%)	
Trans male	3 (0.7%)	0 (0%)	0 (0%)	3 (2.8%)	0 (0%)	0 (0%)	
*Race*							< *0.01***
White	309 (72.0%)	107 (68.6%)	26 (61.9%)	87 (82.1%)	64 (70.3%)	25 (73.5%)	
Black	78 (18.2%)	29 (18.6%)	5 (11.9%)	14 (13.2%)	21 (23.1%)	9 (26.5%)	
All other ace	42 (9.8%)	20 (12.8%)	11 (26.2%)	5 (4.7%)	6 (6.6%)	0 (0.0%)	
*Ethnicity*							< *0.001****
Hispanic	118 (27.5%)	40 (25.6%)	19 (45.2%)	40 (37.7%)	14 (15.4%)	5 (14.7%)	
Non-Hispanic	311 (72.5%)	116 (74.4%)	23 (54.8%)	66 (62.3%)	77 (84.6%)	29 (85.3%)	
*Rank*							0.53
Enlisted	161 (37.5%)	59 (37.8%)	17 (40.5%)	46 (43.4%)	31 (34.1%)	8 (23.5%)	
Officer	199 (46.4%)	77 (49.4%)	20 (47.6%)	42 (39.6%)	44 (48.4%)	16 (47.1%)	
Warrant	69 (16.1%)	20 (12.8%)	5 (11.9%)	18 (17.0%)	16 (17.6%)	10 (29.4%)	
*Military branch*							0.43
Air force	65 (15.2%)	25 (16.0%)	6 (14.3%)	13 (12.3%)	14 (15.4%)	7 (20.6%)	
Army	209 (48.7%)	69 (44.2%)	22 (52.4%)	60 (56.6%)	43 (47.3%)	15 (44.1%)	
Coast guard	49 (11.4%)	20 (12.8%)	3 (7.1%)	10 (9.4%)	12 (13.2%)	4 (11.8%)	
Marine corps	48 (11.2%)	15 (9.6%)	8 (19.0%)	11 (10.4%)	13 (14.3%)	1 (2.9%)	
Navy	58 (13.5%)	27 (17.3%)	3 (7.1%)	12 (11.3%)	9 (9.9%)	7 (20.6%)	
*Education level*							< *0.01***
High school	28 (6.5%)	15 (9.6%)	1 (2.4%)	5 (4.7%)	7 (7.7%)	0 (0.0%)	
AD or some college	169 (39.4%)	52 (33.3%)	12 (28.6%)	50 (47.2%)	42 (46.2%)	13 (38.2%)	
Bachelor’s degree	188 (43.8%)	65 (41.7%)	24 (57.1%)	40 (37.7%)	38 (41.8%)	21 (61.8%)	
Graduate/prof degree	44 (10.3%)	24 (15.4%)	5 (11.9%)	11 (10.4%)	4 (4.4%)	0 (0.0%)	
*Region of station in the U.S.A.*^*a*^							< *0.05**
Midwest	55 (12.8%)	20 (12.8%)	4 (9.5%)	13 (12.3%)	14 (15.4%)	4 (11.8%)	
Northeast	79 (18.4%)	30 (19.2%)	9 (21.4%)	12 (11.3%)	15 (16.5%)	13 (38.2%)	
South	161 (37.5%)	49 (31.4%)	11 (26.2%)	53 (50.0%)	38 (41.8%)	10 (29.4%)	
West	129 (30.1%)	54 (34.6%)	18 (42.9%)	26 (24.5%)	24 (26.4%)	7 (20.6%)	

Notes:

^*a*^States within the U.S. Midwest (IA, IL, IN, KS, MI, MN, MO, ND, NE, OH, SD, WI), Northeast (CT, DC, DE, MA, MD, ME, NH, NJ, NY, PA, RI, VT), Southeast (AL, AR, FL, GA, KY, LA, MS, NC, SC, TN, VA, WV), Southwest (AZ, NM, OK, TX), West (AK, CA, CO, HI, ID, MT, NV, OR, UT, WA, WY)

*Significantly different at significance level of 0.05

**Significantly different at significance level of 0.01

***Significantly different at significance level of 0.001

**Table 2:** Characteristics of the participant’s sexual and health-seeking behaviors and beliefs, segmented by group

Variable	Total (*n* = 429)	Least PrEP Experience [Group 1] (*n* = 156)	Least Comfortable Discussing Sex [Group 2] (*n* = 42)	Most Condomless Sex [Group 3] (*n* = 106)	Less HIV Protection Satisfaction [Group 4] (*n* = 91)	Most PrEP Experience [Group 5] (*n* = 34)	*F*/*χ*^2^ (*p*-value)
*HIRI-MSM risk score *^*b*^							0.08
> = 10	383 (89.3%)	141 (90.4%)	35 (83.3%)	101 (95.3%)	77 (84.6%	29 (85.3%)	
< 10	46 (10.7%)	15 (9.6%)	7 (16.7%)	5 (4.7%)	14 (15.4%)	5 (14.7%)	
*Sex position past 6 months*							< *0.05**
Exclusive/more bottom	155 (36.1%)	57 (36.5%)	11 (26.2%)	44 (41.5%)	28 (30.8%)	15 (44.1%)	
Versatile	155 (36.1%)	48 (30.8%)	15 (35.7%)	46 (43.4%)	35 (38.5%)	11 (32.4%)	
Exclusive/more top	119 (27.7%)	51 (32.7%)	16 (38.1%)	16 (15.1%)	28 (30.8%)	8 (23.5%)	
*# CRAS within last 6 months *^*c*^							< *0.001****
None	69 (16.1%)	18 (11.5%)	15 (35.7%)	8 (7.5%)	19 (20.9%)	9 (26.5%)	
Once/month or less	249 (58.0%)	86 (55.1%)	16 (38.1%)	74 (69.8%)	51 (56.0%)	22 (64.7%)	
Once/week or more	111 (25.9%)	52 (33.3%)	11 (26.2%)	24 (22.6%)	21 (23.1%)	3 (8.8%)	
*Condom use w/regular partner last 6 months*							< *0.05**
Every time	51 (11.9%)	11 (7.1%)	11 (26.2%)	9 (8.5%)	15 (16.5%)	5 (14.7%)	
Often	144 (33.6%)	51 (32.7%)	11 (26.2%)	38 (35.8%)	28 (30.8%)	16 (47.1%)	
Sometimes	111 (25.9%)	37 (23.7%)	10 (23.8%)	31 (29.2%)	26 (28.6%)	7 (20.6%)	
Rarely	68 (15.9%)	25 (16.0%)	6 (14.3%)	14 (13.2%)	17 (18.7%)	6 (17.6%)	
Never	35 (8.2%)	18 (11.5%)	3 (7.1%)	11 (10.4%)	3 (3.3%)	0 (0.0%)	
No regular male partner	20 (4.7%)	14 (9.0%)	1 (2.4%)	3 (2.8%)	2 (2.2%)	0 (0.0%)	
*Condom use w/casual partner last 6 months*							< *0.001****
Every time	46 (10.7%)	20 (12.8%)	13 (31.0%)	9 (8.5%)	4 (4.4%)	0 (0.0%)	
Often	156 (36.4%)	46 (29.5%)	16 (38.1%)	35 (33.0%)	36 (39.6%)	23 (67.6%)	
Sometimes	127 (29.6%)	44 (28.2%)	8 (19.0%)	35 (33.0%)	35 (38.5%)	5 (14.7%)	
Rarely	66 (15.4%)	25 (16.0%)	3 (7.1%)	20 (18.9%)	12 (13.2%)	6 (17.6%)	
Never	10 (2.3%)	4 (2.6%)	0 (0.0%)	3 (2.8%)	3 (3.3%)	0 (0.0%)	
No casual male partner	24 (5.6%)	17 (10.9%)	2 (4.8%)	4 (3.8%)	1 (1.1%)	0 (0.0%)	
*Satisfied w/current level of HIV protection?*							< *0.001****
Satisfied	356 (83.0%)	124 (79.5%)	41 (97.6%)	97 (91.5%)	69 (75.8%)	25 (73.5%)	
Unsatisfied	73 (17%)	32 (20.5%)	1 (2.4%)	9 (8.5%)	22 (24.2%)	9 (26.5%)	
*Previous use or experience with PrEP?*							< *0.001****
Yes	357 (83.2%)	113 (72.4%)	38 (90.5%)	90 (84.9%)	83 (91.2%)	33 (97.1%)	
No	72 (16.8%)	43 (27.6%)	4 (9.5%)	16 (15.1%)	8 (8.8%)	1 (2.9%)	
*Comfort level discussing sex with PCP*							< *0.01***
Extremely uncomfortable	37 (8.6%)	14 (9.0%)	9 (21.4%)	4 (3.8%)	9 (9.9%)	1 (2.9%)	
Somewhat uncomfortable	121 (28.2%)	47 (30.1%)	15 (35.7%)	32 (30.2%)	24 (26.4%)	3 (8.8%)	
Mostly comfortable	209 (48.7%)	70 (44.9%)	15 (35.7%)	59 (55.7%)	43 (47.3%)	22 (64.7%)	
Extremely comfortable	62 (14.5%)	25 (16.0%)	3 (7.1%)	11 (10.4%)	15 (16.5%)	8 (23.5%)	
*Fear of judgement from PCP for being MSM*							< *0.001****
Very fearful	62 (14.5%)	23 (14.7%)	4 (9.5%)	25 (23.6%)	9 (9.9%)	1 (2.9%)	
Somewhat fearful	144 (33.6%)	53 (34.0%)	15 (35.7%)	39 (36.8%)	28 (30.8%)	9 (26.5%)	
Slightly fearful	148 (34.5%)	61 (39.1%)	17 (40.5%)	26 (24.5%)	35 (38.5%)	9 (26.5%)	
Not at all fearful	75 (17.5%)	19 (12.2%)	6 (14.3%)	16 (15.1%)	19 (20.9%)	15 (44.1%)	

Notes:

^*b*^1–47 range. Scores >  = 10 defined as high risk for HIV[54]

^*c*^CRAS (Condomless Receptive Anal Sex)

*Significantly different at significance level of 0.05

**Significantly different at significance level of 0.01

***Significantly different at significance level of 0.001

**Table 3:** Relative importance scores (%) of PrEP program attributes by group

Attributes	Total	Least PrEP Experience [Group 1] (*n* = 156)	Least Comfortable Discussing Sex [Group 2] (*n* = 42)	Most Condomless Sex [Group 3] (*n* = 106)	Less HIV Protection Satisfaction [Group 4] (*n* = 91)	Most PrEP Experience [Group 5] (*n* = 34)
*Dosing method*	45.2%	49.6%	27.7%	47.8%	43.5%	43.4%
*Provider type*	15.8%	14.7%	34.1%	12.3%	14.8%	12.3%
*PrEP visit location*	14.5%	13.7%	19.5%	14.0%	14.3%	14.2%
*Lab evaluation location*	13.4%	12.6%	10.8%	14.3%	14.4%	15.0%
*PrEP dispensing venue*	11.0%	9.5%	8.0%	11.6%	12.9%	15.2%

Notes: Relative importance scores reflect the influence that each attribute has on a participant’s decision-making

**Table 4:** Part-worth utility scores (zero-centered) of PrEP program level choices by group

Attributes	Total	Least PrEP Experience [Group 1] (*n* = 156)	Least Comfortable Discussing Sex [Group 2] (*n* = 42)	Most Condomless Sex [Group 3] (*n* = 106)	Less HIV Protection Satisfaction [Group 4] (*n* = 91)	Most PrEP Experience [Group 5] (*n* = 34)
*Dosing method*						
Daily tablet	21.75	3.82	− 22.49	98.38	20.41	− 76.70
On-demand	8.99	− 41.95	20.49	38.60	76.59	− 44.77
Rectal douche	− 60.37	− 111.74	− 47.37	− 109.24	27.01	77.78
PrEP injection	15.58	71.08	2.09	− 0.88	− 47.62	− 1.89
PrEP implant	14.05	78.78	47.28	− 26.86	− 76.39	45.58
*Provider type*						
Military	5.55	24.90	− 85.15	14.00	11.81	− 14.34
Civilian	− 5.55	− 24.90	85.15	− 14.00	− 11.81	14.34
*PrEP visit location*						
On-base	2.45	− 0.37	− 56.87	22.59	13.91	− 4.84
Off-base	− 10.13	− 16.28	32.18	− 18.08	− 16.92	8.77
Smartphone	7.69	16.65	24.69	− 4.51	3.01	− 3.92
*Lab evaluation location*						
On-base	12.65	13.22	− 18.01	22.86	14.27	11.76
Off-base	− 9.68	− 15.24	10.39	− 13.64	− 8.40	− 0.07
Mail-in kit	− 2.97	2.02	7.61	− 9.22	− 5.87	− 11.69
*PrEP dispensing venue*						
On-base	12.66	13.78	− 8.98	19.45	11.13	17.12
Off-base	− 8.42	− 9.73	5.39	− 16.37	− 8.31	4.97
Mail delivery	− 4.23	− 4.05	3.59	− 3.08	− 2.82	− 22.09
*None*^*d*^	− 54.69	− 52.32	60.64	− 137.59	− 34.36	− 4.02

Notes:

Zero-centered part-worth utility scores imply the positive or negative magnitude of the preference for the level choice in relation to the other level options within the same attribute

^*d*^ “None” denotes the magnitude in which an individual is not willing to take PrEP in any scenario (i.e., a negative value in “None” represents the magnitude that an individual IS willing to take PrEP in a particular scenario)

**Table 5:** Description of hypothetical PrEP scenarios with different attributes and levels

PrEP scenario ^*e*^		PrEP attributes and level options				
		Dosing method	Provider type	Visit location	Lab evaluation	Dispensing venue
2	On-Base Military Daily Tablet	Daily Tablet	Military	On-base	On-base	On-base
	Smartphone Military Daily Tablet	Daily Tablet	Military	Smartphone	On-base	On-base
3	Smartphone Military On-Demand	On-Demand	Military	Smartphone	On-Base	On-Base
4	Smartphone Military Injection	PrEP Injection	Military	Smartphone	On-base	On-base
5	Smartphone Military Implant	PrEP Implant	Military	Smartphone	On-base	On-base
6	Off-Base Civilian Daily Tablet	Daily Tablet	Civilian	Off-Base	Off-Base	Off-Base
7	Off-Base Civilian Implant	PrEP Implant	Civilian	Off-Base	Off-Base	Off-Base
8	Off-Base Civilian Rectal PrEP	Rectal Douche	Civilian	Off-Base	Off-Base	Off-Base

Notes:

^*e*^Scenarios descriptions reference Scenarios 1 through 8 in Table 6

**Table 6:** Program interest (share of preference) rates (%) of hypothetical PrEP scenarios by group

PrEP scenario ^*f*^		Total population and group PrEP program interest scores					
		Total	Least PrEP Experience [Group 1] (*n* = 156)	Least Comfortable Discussing Sex [Group 2] (*n* = 42)	Most Condomless Sex [Group 3] (*n* = 106)	Less HIV Protection Satisfaction [Group 4] (*n* = 91)	Most PrEP Experience [Group 5] (*n* = 34)
2	On-Base Military Daily Tablet	66.4%	71.1%	2.9%	92.2%	69.1%	35.4%
	Smartphone Military Daily Tablet	69.6%	79.2%	7.5%	93.0%	67.5%	35.6%
3	Smartphone Military On-Demand	67.6%	67.6%	9.9%	89.1%	77.6%	45.0%
4	Smartphone Military Injection	69.6%	90.9%	9.4%	83.8%	51.2%	50.8%
5	Smartphone Military Implant	68.5%	91.3%	13.8%	80.5%	44.0%	59.4%
6	Off-Base Civilian Daily Tablet	57.7%	48.9%	63.9%	82.6%	49.1%	36.3%
7	Off-Base Civilian Implant	59.3%	67.7%	84.9%	59.9%	29.6%	67.1%
8	Off-Base Civilian Rectal PrEP	40.5%	21.2%	61.5%	42.7%	49.3%	72.9%

Notes:

Share of preference denotes the percent of respondents that would prefer or have an interest in the respective PrEP delivery program scenario with a particular combination of program attributes based on part-worth utilities obtained during the conjoint analysis survey

^*f*^Descriptions of PrEP Scenarios 1 through 8 are explained in Table 5

**Table 7:** Acceptability (mean) of PrEP delivery program groupings with multiple scenario options

PrEP program group offering ^*g*^	Total population and group PrEP program interest scores					
	Total	Least PrEP Experience [Group 1] (*n* = 156)	Least Comfortable Discussing Sex [Group 2] (*n* = 42)	Most Condomless Sex [Group 3] (*n* = 106)	Less HIV Protection Satisfaction [Group 4] (*n* = 91)	Most PrEP Experience [Group 5] (*n* = 34)
PrEP Program Grouping #1						
**I**: On-Base Military Daily Tablet	**34.8%**	33.5%	0.3%	53.7%	38.2%	15.9%
**II.** Smartphone Military Daily Tablet	**25.0%**	35.2%	2.5%	25.1%	21.2%	15.7%
**III**: Off-Base Civilian Daily Tablet	**21.3%**	14.5%	62.1%	17.9%	18.2%	21.3%
**Sample total:**	***81.1%***	*83.2%*	*64.9%*	*96.7%*	*77.6%*	*52.9%*
PrEP Program Grouping #2						
**I**: On-Base Military Daily Tablet	**23.9%**	11.4%	0.2%	50.1%	32.3%	5.5%
**II.** Smartphone Military Daily Tablet	**8.9%**	2.9%	0.4%	18.8%	14.1%	2.1%
**III**: Off-Base Civilian Daily Tablet	**11.0%**	3.4%	19.9%	15.9%	15.9%	5.8%
**IV**: Smartphone Military Injection	**15.3%**	29.7%	1.5%	5.3%	8.8%	14.5%
**V**: Smartphone Military Implant	**17.9%**	36.4%	2.8%	4.9%	6.0%	23.5%
**VI:** Off-Base Civilian Implant	**13.6%**	11.6%	60.5%	1.8%	3.0%	29.6%
**Sample total:**	***90.6%***	*95.4%*	*85.3%*	*96.8%*	*80.1%*	*81.0%*

Notes:

^*g*^Descriptions of PrEP Scenarios are explained in Table 5

## Code Availability

All data analyses were performed using XLSTAT and Sawtooth Lighthouse Studio 9.0.

## Abbreviations

PrEP: Pre-exposure prophylaxis
MSM: Men who have sex with men
HIV: Human immunodeficiency virus
LGBT: Lesbian, gay, bi and transgender
HB: Hierarchical Bayes
ACBC: Adaptive choice-based conjoint
HIRI-MSM: HIV Incidence Risk Index for men who have sex with men
IP: Internet protocol
RLH: Root likelihood
PWUS: Part-worth utility scores
RIS: Relative importance score
PCP: Primary care provider
